# Direct Determination of Ni^2+^-Capacity of IMAC Materials Using Near-Infrared Spectroscopy

**DOI:** 10.3390/molecules23123072

**Published:** 2018-11-24

**Authors:** Christian G. Kirchler, Raphael Henn, Julia Modl, Felix Münzker, Tanja H. Baumgartner, Florian Meischl, Alexander Kehle, Günther K. Bonn, Christian W. Huck

**Affiliations:** 1 Institute of Analytical Chemistry and Radiochemistry, CCB-Center for Chemistry and Biomedicine, Innrain 80/82, 6020 Innsbruck, Austria; Christian.Kirchler@uibk.ac.at (C.G.K.); Raphael.Henn@uibk.ac.at (R.H.); Julia.Modl@student.uibk.ac.at (J.M.); Felix.Muenzker@student.uibk.ac.at (Fe.M.); baumgartner_tanja@t-online.de (T.H.B.); Florian.Meischl@uibk.ac.at (Fl.M.); alexander.kehle@student.uibk.ac.at (A.K.); Guenther.Bonn@uibk.ac.at (G.K.B.); 2 ADSI—Austrian Drug Screening Institute, Innrain 66a, 6020 Innsbruck, Austria

**Keywords:** near-infrared spectroscopy, IMAC, partial least squares regression, Ni^2+^-capacity, method validation

## Abstract

The present paper reports a new method for the quantification of the Ni^2+^-capacity of an immobilized metal affinity chromatography (IMAC) material using near-infrared spectroscopy (NIRS). Conventional analyses using UV absorption spectroscopy or atomic absorption spectrometry (AAS) need to dissolve the silica-based metal chelate sorbent as sample pretreatment. In the first step, those methods were validated on the basis of an ideal homogenous NiSO_4_-solution and unveiled that UV with an intermediate precision of 2.6% relative standard deviation (RSD) had an advantage over AAS with an intermediate precision of 6.5% RSD. Therefore, UV analysis was chosen as reference method for the newly established NIRS model which has the advantage of being able to measure the material directly in diffuse reflection mode. Partial least squares regression (PLSR) analysis was used as multivariate data analysis tool for quantification. The best PLSR result obtained was: coefficient of determination (R^2^) = 0.88, factor = 2, root mean square error of prediction (RMSEP) = 22 µmol/g (test-set validation) or 7.5% RSD_PLSR_. Validation of the Ni^2+^-capacity using UV absorption spectroscopy resulted in an intermediate precision of ±18 µmol/g or 5.0% RSD. Therefore, NIRS provides a fast alternative analysis method without the need of sample preparation.

## 1. Introduction

The affinity of proteins to transition elements such as zinc, copper, nickel, etc. in their ionic state bonded to a stationary phase was described in 1975 by Porath et al. for the first time [1]. Based on this knowledge further investigations were carried out on purification and analysis of proteins using this technique, which is nowadays well known as immobilized metal affinity chromatography (IMAC) [2,3,4,5,6]. Ni^2+^ on an IMAC material based on a nitrilotriacetic acid adsorbent is found to bind specific to proteins which contain neighboring histidine residues [7]. This approach is the fundament for the purification of proteins due to their histidine content [8]. Therefore, six attached histidine residues (HisTag) are common as affinity tag and result in a high purification of the proteins in high yield [9,10].

Typically, the immobilized metal ion content regarding the capacity of the IMAC material is determined by UV absorption spectroscopy or atomic absorption spectrometry (AAS). For that, the analysis is in need of sample preparation to release the metal ions in the first step and to quantify them in the second step [11,12].

Near-infrared spectroscopy (NIRS) is known to be a fast analytical technique which operates noninvasively and usually needs no sample preparation. This vibrational spectroscopic technique is based on the interaction of light with matter, where light in the wavenumber region from 12,500 to 4000 cm^−1^ (800–2500 nm) is used [13]. The near-infrared (NIR) region consist of three regions whose borders are not rigid. In region I (12,500–8500 cm^−1^), which is also known as short-wave NIR (SWNIR) or Herschel region, bands emerge not only due to overtones and combination modes but also due to electronic transitions. In region II (8500–5500 cm^−1^), bands due to first overtones of stretching vibrations and combination modes can be observed. In region III (5500–4000 cm^−1^), bands due to combination modes arise [14]. In the NIR region, mainly C–H, O–H and N–H moieties cause the absorption based on the anharmonic character of the vibration modes and the change of the dipole moment during the vibration [15]. The complex NIR spectrum contains chemical as well as physical information. Therefore, in NIRS, usually chemometrics or multivariate data analysis (MVA) is applied to extract the specific information of interest [16]. The most common method for multivariate regression, which was also applied in this study, is partial least squares regression (PLSR) [17]. Broadly speaking, the very likely successful establishment of a NIRS analysis needs at least an analyte concentration of 0.1% [13]. In the present study, a novel time- and resource-saving NIRS method for the quantification of the Ni^2+^-capacity of IMAC materials, which are based on the synthesis postulated by Anspach [18], was established and compared to the common UV absorption spectroscopic method.

## 2. Results and Discussion

### 2.1. IMAC Materials

The repeated syntheses of the IMAC materials showed that the resulting Ni^2+^-capacity was not reproducible. The minimum difference by using the same silica gels as starting materials was 50 µmol/g and went up to a maximum difference of 138 µmol/g of the Ni^2+^-capacity. Therefore, 32 IMAC materials differing in Ni^2+^-capacity were synthesized by reapplying 12 silica gels. The usage of different pore and particle size ensured that a variation of these physical parameters which strongly affect the NIR spectra was taken into account. Correlation analysis of the Ni^2+^-capacity and pore size gave a Pearson correlation coefficient [19] of −0.6 and therefore showed a moderate negative correlation. No correlation analysis was done for the particle size as just a range was specified by the producers. The Ni^2+^-capacity of all the IMAC materials was in the range from 156 to 444 µmol/g. Details of all 32 IMAC materials are given in Table S1 in the Supplementary Materials.

### 2.2. UV and AAS Method Validation

Two techniques are typically used for the quantification of the Ni^2+^-capacity of IMAC materials: UV absorption spectroscopy and AAS [11,12]. The first goal of this study was to identify the quantification method which is better suited as reference analysis for the establishment of a PLSR model on the basis of NIRS. As the precision of the reference values limits the possible performance of the PLSR model, the repeatability and the intermediate precision were used as decision criteria to determine the better suited reference analysis. In the first step, the IMAC materials were not used for this determination as they need an additional sample preparation step which takes an additional error into account and therefore was determined after (Section 2.3). Three solutions containing low, middle and high nickel concentration referred to the range of the calibration were prepared and used for the determination of the repeatability and intermediate precision. For the intermediate precision calibration, solutions were prepared freshly for each of the five measurement days but were used four times within a day for the repeatability. The coefficients of determination (R^2^), limits of detection (LOD) and limits of quantification (LOQ) for the calibrations were 0.9949 ≤ R^2^ ≤ 0.9998, 0.00028 ≤ LOD ≤ 0.0014 mol/L and 0.0010 ≤ LOQ ≤ 0.0047 mol/L for the UV method and 0.9821 ≤ R^2^ ≤ 0.9972, 5.6 × 10^−6^ ≤ LOD ≤ 1.4 × 10^−5^ mol/L and 1.9 × 10^−5^ ≤ LOQ ≤ 4.7 × 10^−5^ mol/L for the AAS method, respectively. UV measurements resulted in a repeatability of 1.9% relative standard deviation (RSD) and intermediate precision of 2.6% RSD. AAS measurements resulted in a repeatability of 6.2% RSD and intermediate precision of 6.5% RSD. The results unveiled that the UV absorption spectroscopy method performed better than the AAS one and therefore was better suited for the determination of the Ni^2+^-capacity of the IMAC materials. The raw data and regression parameters for all nickel solution measurements and calibrations are given in Tables S2–S17 in the Supplementary Materials.

### 2.3. Reference Analysis Method Validation

In the second step, the performance of the Ni^2+^-capacity reference analysis for creating the PLSR model was evaluated. Therefore, six IMAC materials were synthesized for the determination of the repeatability and the intermediate precision. The R^2^, LOD and LOQ of all calibrations were 0.9977 ≤ R^2^ ≤ 0.9998, 0.00028 ≤ LOD ≤ 0.0010 mol/L and 0.00092 ≤ LOQ ≤ 0.0032 mol/L, respectively. The Ni^2+^-capacity reference analysis gave a repeatability of 4.2% RSD or ±15 µmol/g and an intermediate precision of 5.0% RSD or ±18 µmol/g. In contrast to the UV method validation of the standard solutions which had an intermediate precision of 2.6% RSD (Section 2.2), the reference analysis resulted in a worse precision. This was caused by the additional sample preparation step in which the nickel had to be released from the IMAC material. Furthermore, handling of solid materials takes possible inhomogeneity into account, which also contributes to the lower precision of the analysis. The raw data and regression parameters for all precision measurements of the reference analysis are given in Tables S18–S23 in the Supplementary Materials.

### 2.4. Multivariate Data Analysis of NIR Spectra

**Figure 1:**
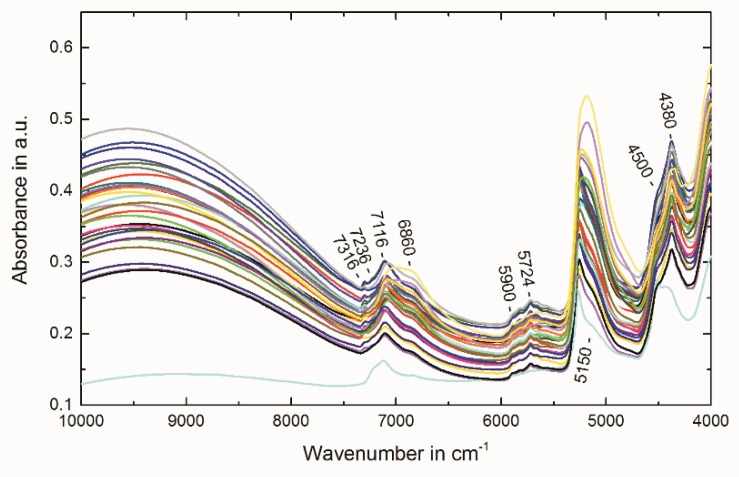
Raw NIR spectra of all 32 IMAC materials.

**Figure 2:**
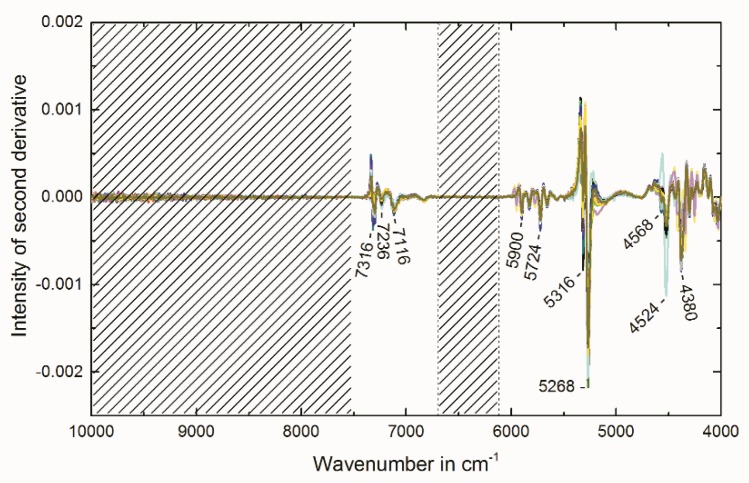
Pretreated NIR spectra of all 32 IMAC materials (Savitzky–Golay derivative order: 2, polynomial order: 2, 5 smoothing points).

Raw NIR spectra of all 32 IMAC materials used for MVA are shown in Figure 1. The band assignment was done by using the raw NIR spectra (Figure 1) and the second derivative (Figure 2). The latter one enhances spectral features and therefore resolves small bands. This means that a single peak in a zeroth order spectrum appears as negative band with its minimum at the same wavenumber as the maximum in the absorption band [20]. The broad band with its maximum at 9500 cm^−1^ appears after nickel ion immobilization. This signal can be assigned to the electronic transition of the nickel complex but is not due to overtone or combination vibration [21,22]. The bands at 7316 cm^−1^ and 7300 cm^−1^ observed through the analysis of the second derivative arise from the first overtone of the OH stretching vibrations of the free silanol groups (literature: 7316 cm^−1^) [20,23,24,25]. The band at 7236 cm^−1^ can be assigned to silanol groups hydrogen bonded to water molecules (literature: 7225 cm^−1^) [20]. The band at 7116 cm^−1^ is due to water molecules hydrogen bonded to silanol groups and water molecules hydrogen bonded to other water molecules (literature: 7121 cm^−1^) [20]. The broad band at 6860 cm^−1^ (literature: ca. 6900 cm^−1^ [26] and 6861 cm^−1^ [20]) is due to combination involving the symmetric and asymmetric stretching modes of the water molecule [26]. The bands at 5900, 5828, 5724 and 5660 cm^−1^ in region from 6000 to 5600 cm^−1^ can be assigned to overtones of CH stretching vibrations [26]. Analysis of the second derivative (Figure 2) reveals a band at 5316 which is due the water molecules adsorbed onto the free silanol groups on the surface (literature: 5314 cm^−1^) and a band at 5268 cm ^−1^ due to the water molecules adsorbed onto hydrogen bonded silanol groups (literature: 5270 cm^−1^) [20,23]. The broad band, which appears at 5150 cm^−1^, can be assigned to the combination of the asymmetric stretch and bending of the water molecule (literature 5150 cm^−1^) [26]. The bands at about 4500 cm^−1^ in the raw spectra or seen at 4568 and 4524 cm^−1^ in the second derivative represent the combination band of OH stretch with one of the SiO_2_ fundamentals (literature: 4450 and 4520 cm^−1^ [26] or 4579, 4518 and 4422 cm^−1^ [20]). The bands in the region below 4400 cm^−1^ can be assigned to the first combination region of CH [26].

**Figure 3:**
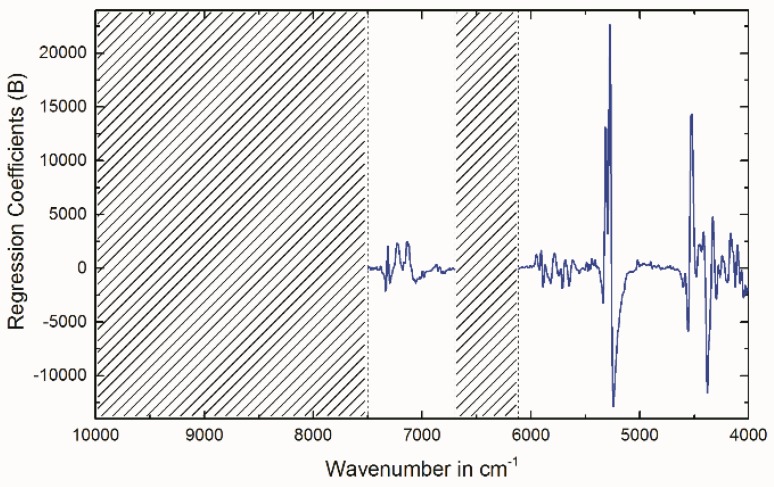
Regression coefficients plot (Factor 2) of the established PLSR model.

The best PLSR model was obtained by applying the following spectral pre-treatment: second derivative (Savitzky–Golay, polynomial order: 2, number of smoothing points: 5). Wavenumber regions used for the model were: 7500–6700 cm^−1^ and 6120–4008 cm^−1^. Figure 2 illustrates the pretreated spectra and the removed regions (indicated by the crossed-out areas). The region from 10,000 to 7504 cm^−1^ was kept out since the very broad band that can be found in this region in the raw spectra (Figure 1) gives no signal in the second derivative spectra (Figure 2). This means that this band is unsuitable for the calculation of a multivariate PLSR model. The region 6696–6124 cm^−1^ only contains the foothills of broad bands. Therefore, no defined signals can be observed in the second derivative spectra. The results of the established PLSR model are depicted in Table 1. The regression coefficients for factor 2 which summarize the relationship between the wavenumbers and the Ni^2+^-capacity are plotted in Figure 3. By comparing the second derivative spectra in Figure 2 to the regression coefficients in Figure 3, it can be concluded that the observed regression coefficients correspond to the observed molecular vibrations described above. As the root mean square error of prediction (RMSEP) shows the best estimation for the future performance of the PLSR model [13], this quality parameter should be used for the direct comparison of the reference analysis and the introduced PLSR model. Due to the recording of the NIR spectra and the measurement of the reference values at different days, the RMSEP of 22 µmol/g (7.5% RSD_PLSR_) regarding the Ni^2+^-capacity represents the intermediate precision of the NIRS method (including the PLSR model, NIRS measurements and MVA). The UV absorption spectroscopy method used as Ni^2+^-capacity reference analysis achieved an intermediate precision of ±18 µmol/g (5.0% RSD). Therefore, the NIRS model performed comparable. Raw spectra, reference data and the established PLSR models are provided in a Microsoft Excel File and The Unscrambler X File in the Supplementary Materials.

## 3. Materials and Methods

### 3.1. Chemicals

3-Glycidyloxypropyl)trimethoxysilane (GLYMO, ≥98%) and iminodiacetic acid (IDA, 98%) were obtained from Sigma-Aldrich (Buchs, Switzerland). Ethylenediaminetetraacetic acid disodium salt dihydrate (EDTA-Na_2_, ≥99%) was purchased from Fluka (Buchs, Switzerland). Nickel(II) sulfate hexahydrate (NiSO_4_·6H_2_O, ACS), sodium hydroxide pellets (NaOH, >99%) and sodium chloride (NaCl, ACS) were purchased from Merck (Darmstadt, Germany). Hydrochloric acid (HCl, Supra 35%) was ordered from Carl Roth (Karlsruhe, Germany). Deionized water was purified by Milli-Q^®^ Reference water purification system (Merck Millipore, Darmstadt, Germany) and was specified by an electrical resistivity of 18.2 MΩcm.

Gas supply for AAS: Acetylene 2.6 (>99.6 vol%, Messer Austria, Gumpoldskirchen, Austria); compressed air.

The following 12 silica gels differing in pore and particle size were gained: Carl Roth (Karlsruhe, Germany), 35 Å (40–63 µm), 60 Å (20–45 µm), 60 Å (30–200 µm), 60 Å (35–70 µm), 60 Å (40–63 µm), 60 Å (200–500 µm), 150 Å (35–70 µm), 150 Å (70–200 µm), 1000 Å (35–70 µm), and 250 Å (40–63 µm); Fluka (Buchs, Switzerland), 60 Å (63–200 µm); and Sigma-Aldrich (Buchs, Switzerland), 100 Å (63–200 µm).

### 3.2. Synthesis

#### 3.2.1. Activation of Silica Gel

First, 0.56 g (14 mmol) NaOH were dissolved into 28 mL H_2_O. Then, 3 g silica gel were added to the solution and stirred for 30 min at room temperature. Next, 2.3 mL 6 M HCl (14 mmol) were used to neutralize the solution. Afterwards, the silica gel was filtered and washed several times with H_2_O. The particles were dried at 45 °C under vacuum (300 mbar).

#### 3.2.2. Preparation of GLYMO-IDA

A solution of 29.4 g NaOH and 19.6 g (144 mmol) iminodiacetic acid (IDA) in 210 mL H_2_O was prepared at 0 °C. Thirty-five grams (144 mmol) of 3-glycidyloxypropyl)trimethoxysilane (GLYMO) were slowly added during 30 min. The mixture was allowed to warm up to room temperature and was stirred for 4 h. Afterwards, the temperature was raised to 65 °C and the reaction mixture was stirred for 18 h [18].

#### 3.2.3. Immobilization of GLYMO-IDA on the Silica Gel

Nine milliliters of 6 M HCl were added to 24 mL of the GLYMO-IDA solution to adjust the pH-value to 3.5. The solution was diluted with 50 mL H_2_O and 2 g of activated silica gel were added. The suspension was refluxed at 100 °C for 3 h. The mixture was allowed to cool down to room temperature. The particles were filtered, washed several times with H_2_O and dried at 45 °C under vacuum (300 mbar) [18].

#### 3.2.4. Nickel Ion Immobilization

**Figure 4:**
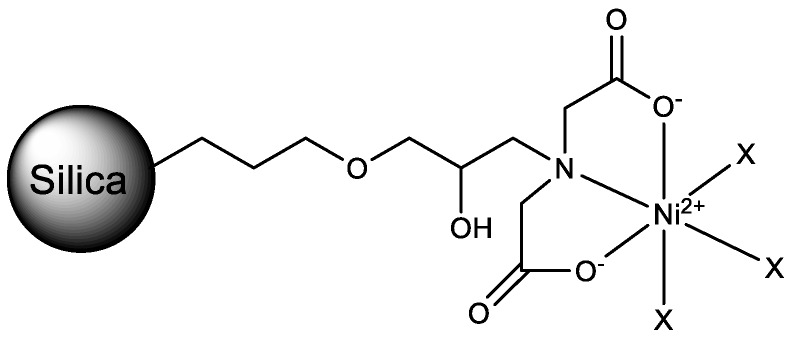
Structure of the IMAC material [6]. X, available orbitals for electron donors (water, imidazole, etc.).

Forty milliliters of 0.1 M nickelsulfate solution were added to 1 g of the silica-GLYMO-IDA material. The suspension was stirred for 2 h at room temperature. To remove unbound nickel ions, the particles were filtered and washed with H_2_O, 0.5 M NaCl solution and H_2_O successively. The obtained turquoise IMAC material was dried at 45 °C under vacuum (300 mbar). The structure [6] is shown in Figure 4.

### 3.3. UV Absorption Spectroscopy

First, 1.5 mL 0.05 M EDTA solution were added to 100 mg of the IMAC material and mixed for 10 min at 1600 rpm using a Thermomixer C (Eppendorf, Hamburg, Germany). After centrifugation for 5 min at 13,200 rpm or 15,000 rcf using a Centrifuge 5418 (Eppendorf, Hamburg, Germany), the supernatant was removed and the procedure was repeated. The supernatants were combined and filled up to 5 mL using a volumetric flask. The solution was transferred into a cuvette and the absorption was measured in triplicate at λ = 380 nm using a Jenway Genova Plus Life Science Spectrophotometer (Cole-Parmer, Stone, UK). External calibration was done by dissolving nickel sulfate hexahydrate in 0.05 M EDTA solution.

### 3.4. Atomic Absorption Spectrometry

A Perkin Elmer AAnalyst 800 (Perkin Elmer, Waltham, MA, USA) equipped with the burner system and a Nickel (Ni) Lumina Hollow Cathode Lamp Series N3050152 (Perkin Elmer, Waltham, MA, USA) was used for AAS. The system was controlled with the software WinLab32 for AA (Perkin Elmer, Waltham, MA, USA). Parameters of the used method: Flame-Oxidant, Air; Oxidant Flow, 17.0 L/min; Acetylene Flow, 2.0 L/min; Spectrometer-Element, Ni; Lamp Current, 30 mA; Wavelength, 232.0 nm; Slit width, 0.2 H nm; Signal-Type, AA; Measurement, time average; Read Parameters-Time, 3.0 s; Delay Time, 0 s; Replicates, 3.

The solutions used for UV absorption spectroscopy were diluted with 0.05 M EDTA to a nickel concentration of approximately 0.14 mM or 8 µg/mL using a 50 mL volumetric flask to be in the range of the calibration (2–15 µg/mL). Measurements were done in triplicate. External calibration was done by dissolving nickel sulfate hexahydrate in 0.05 M EDTA solution.

### 3.5. Near-Infrared Spectroscopy

Measurements of the NIR spectra of all 32 samples were done using the NIRFlex N-500 FT-NIR spectrometer (Büchi, Flawil, Switzerland) equipped with the Solids Cell N500-001 and the Vial Add-on N510-002, which sequentially scans 6 samples. The operating software was NIR Ware 1.4.3010 (Büchi, Flawil, Switzerland). Spectra were recorded in diffuse reflection in the wavenumber region from 10,000 to 4000 cm^−1^ accumulating 64 scans with a spectral resolution of 8 cm^−1^ and a digital resolution of 4 cm^−1^. Sample measurements were done in triplicate and averaged to one representative spectrum for each sample.

### 3.6. UV and AAS Method Validation

The precision of the UV absorption spectroscopy and AAS methods were evaluated by determination of the repeatability (also termed intra-assay precision) and intermediate precision referred to ICH guidelines [27,28]. Therefore, three solutions with low, mid and high nickel concentrations (11, 23 and 34 mM for UV absorption spectroscopy and diluted to 4, 8, and 11 µg/mL for AAS, respectively) were prepared using nickel sulfate hexahydrate in 0.05 M EDTA. The measurements were carried out four times per day for five days. External calibration was done for each measurement. Six Calibration standards for UV absorption spectroscopy (5, 13, 21, 29, 37 and 45 mM) and AAS (2, 5, 7, 9, 12 and 15 µg/mL) were prepared freshly each day. Calculations of LOD and LOQ were executed according to DIN32645: 2008-11 [29]. Experimental data for all measurements are given in Tables S2–S17 in the Supplementary Materials.

### 3.7. Reference Analysis Method Validation

Method validation of the precision of the IMAC material Ni^2+^-capacity determination was done by using UV absorption spectroscopy which showed a better performance than AAS (Section 2.2 and Section 2.3). Therefore, six IMAC materials were synthesized. Repeatability and intermediate precision were determined. The measurements were carried out three times per day for three days. Calibration solutions were prepared freshly on each of the three measurement days but were used three times within a day. External calibration was done as described in Section 3.6. Calculations of LOD and LOQ were executed according to DIN32645: 2008-11 [29]. Experimental data are given in Tables S18–S23 in the Supplementary Materials.

### 3.8. Multivariate Data Analysis

MVA was carried out by using the software The Unscrambler X Version: 10.5 (CAMO Software, Oslo, Norway). Since the measured spectra were recorded in reflectance, all spectra had to be transformed to absorbance units to ensure linear relation between spectral and reference data. Furthermore, spectra were pretreated by multiplicative scatter correction (MSC) [30] or standard normal variate (SNV) transformation [31] and first or second derivative. All spectral pretreatments were tested alone and in combination. Derivatives were carried out by Savitzky–Golay differentiation [32] with a quadratic polynomial and optimization of the applied smoothing points (SP). PLSR models were calculated with the NIPALS algorithm [33] and UV absorption spectroscopy data as reference analysis. Spectral regions without relevant information were kept out. Validation was carried out by test-set validation (TSV) and full cross validation (CV) also known as leave one out cross validation (LOOCV) [33]. TSV was done as follows: Two thirds of the IMAC materials (21 samples, distributed representatively over the nickel concentration range and including extreme values) were used for calibration. The remaining third (11 samples) were used as test-set. Evaluation of the calculated PLSR models was carried out by observation of following parameters: root mean square error of calibration (RMSEC), root mean square error of cross validation (RMSECV) for CV, root mean square error of prediction (RMSEP) for TSV, coefficient of determination (R^2^), regression coefficients, the number of factors and the relative standard deviation (RSD) calculated as follows: % RSD_PLSR_ = (RMSECV or RMSEP/Mean value of the calibration range) × 100%.

## 4. Conclusions

UV absorption spectroscopy was unveiled to be superior to AAS for the quantification of the Ni^2+^-capacity of the synthetized IMAC materials. This method was selected as reference analysis for the establishment of a PLSR model using NIRS in hyphenation with MVA. The NIRS model performed almost as well as the UV reference analysis and better than the AAS method. Therefore, the analysis using NIRS represents an attractive alternative for the determination of the Ni^2+^-capacity of IMAC materials as it operates noninvasively, fast and needs no sample preparation.

## Figures and Tables

**Table 1:** Parameters of the established PLSR model.

	CV	TSV
RMSEC in µmol/g	21	21
RMSECV or RMSEP in µmol/g	24	22
R^2^_calibration_	0.90	0.90
R^2^_validation_	0.88	0.88
% RSD_PLSR_	8.0	7.5
Factor	2	2
Calibration range in µmol/g	156–444	

Abbreviations: full cross validation (CV), test-set validation (TSV), root mean square error of calibration (RMSEC), root mean square error of cross validation (RMSECV) for CV, root mean square error of prediction (RMSEP) for TSV, coefficient of determination (R^2^), relative standard deviation (RSD).

## Supplementary Materials

The following are available online, Table S1: List of synthesized IMAC materials, Table S2: Calibration data for method validation of UV absorption spectroscopy Day 1, Table S3: Calibration data for method validation of UV absorption spectroscopy Day 2, Table S4: Calibration data for method validation of UV absorption spectroscopy Day 3, Table S5: Calibration data for method validation of UV absorption spectroscopy Day 4, Table S6: Calibration data for method validation of UV absorption spectroscopy Day 5, Table S7: Measured sample concentrations of the method validation of UV absorption spectroscopy Day 1–2, Table S8: Measured sample concentrations of the method validation of UV absorption spectroscopy Day 3–4, Table S9: Measured sample concentrations of the method validation of UV absorption spectroscopy Day 5, Table S10: Calibration data for method validation of AAS Day 1, Table S11: Calibration data for method validation of AAS Day 2, Table S12: Calibration data for method validation of AAS Day 3, Table S13: Calibration data for method validation of AAS Day 4, Table S14: Calibration data for method validation of AAS Day 5, Table S15: Measured sample concentrations of the method validation of AAS Day 1–2, Table S16: Measured sample concentrations of the method validation of AAS Day 3–4, Table S17: Measured sample concentrations of the method validation of AAS Day 5, Table S18: Calibration data for method validation of the Ni^2+^-capacity determination by UV absorption spectroscopy Day 1, Table S19: Calibration data for method validation of the Ni^2+^-capacity determination by UV absorption spectroscopy Day 2, Table S20: Calibration data for method validation of the Ni^2+^-capacity determination by UV absorption spectroscopy Day 3, Table S21: Measured Ni^2+^-capacity of the method validation by UV absorption spectroscopy Day 1, Table S22: Measured Ni^2+^-capacity of the method validation by UV absorption spectroscopy Day 2, Table S23: Measured Ni^2+^-capacity of the method validation by UV absorption spectroscopy Day 3, Microsoft Excel File: Raw spectra and reference data, The Unscrambler X File: PLSR Supplementary Materials—The Unscrambler.

## References

[1] Porath J., Carlsson J., Olsson I., Belfrage G. (1975). Metal chelate affinity chromatography, a new approach to protein fractionation. Nature.

[2] El Rassi Z., Horváth C. (1986). Metal chelate-interaction chromatography of proteins with iminodiacetic acid-bonded stationary phases on silica support. J. Chromatogr. A.

[3] Belew M., Porath J. (1990). Immobilized metal ion affinity chromatography: Effect of solute structure, ligand density and salt concentration on the retention of peptides. J. Chromatogr. A.

[4] Kato Y., Nakamura K., Hashimoto T. (1986). High-performance metal chelate affinity chromatography of proteins. J. Chromatogr. A.

[5] Belew M., Yip T.T., Andersson L., Ehrnström R. (1987). High-performance analytical applications of immobilized metal ion affinity chromatography. Anal. Biochem..

[6] Sulkowski E. (1985). Purification of proteins by imac. Trends Biotechnol..

[7] Hochuli E., Döbeli H., Schacher A. (1987). New metal chelate adsorbent selective for proteins and peptides containing neighbouring histidine residues. J. Chromatogr. A.

[8] Zachariou M. (2008). Affinity Chromatography: Methods and Protocols.

[9] Bornhorst J.A., Falke J.J. (2000). Purification of proteins using polyhistidine affinity tags. Methods Enzym..

[10] Trojer L., Stecher G., Feuerstein I., Lubbad S., Bonn G. (2005). Characterisation and evaluation of metal-loaded iminodiacetic acid–silica of different porosity for the selective enrichment of phosphopeptides. J. Chromatogr. A.

[11] Trojer L., Stecher G., Feuerstein I., Bonn G. (2005). Cu(ii)-loaded iminodiacetic acid-silica particles for protein profiling of human serum samples using surface-enhanced affinity capture: Support porosity effects. Rapid Commun. Mass Spectrom..

[12] Wu C.-Y., Suen S.-Y., Chen S.-C., Tzeng J.-H. (2003). Analysis of protein adsorption on regenerated cellulose-based immobilized copper ion affinity membranes. J. Chromatogr. A.

[13] Burns D.A., Ciurczak E.W. (2007). Handbook of Near-Infrared Analysis.

[14] Ozaki Y. (2012). Near-infrared spectroscopy—Its versatility in analytical chemistry. Anal. Sci..

[15] Siesler H.W., Ozaki Y., Satoshi K., Michael H.H. (2002). Near-Infrared Spectroscopy: Principles, Instruments, Applications.

[16] Chalmers J.M., Griffiths P.R. (2001). Handbook of Vibrational Spectroscopy.

[17] Kessler W. (2007). Multivariate Datenanalyse für die pharma-, bio- und prozessanalytik: Ein lehrbuch.

[18] Anspach F.B. (1994). Silica-based metal chelate affinity sorbents i. Preparation and characterization of iminodiacetic acid affinity sorbents prepared via different immobilization techniques. J. Chromatogr. A.

[19] Pearson K. (1895). Note on regression and inheritance in the case of two parents. Proc. R. Soc. Lond..

[20] Christy A.A. (2010). New insights into the surface functionalities and adsorption evolution of water molecules on silica gel surface: A study by second derivative near infrared spectroscopy. Vib. Spectrosc..

[21] Blakeley R.T., Dixon N.E., Zerner B. (1983). Jack bean urease vii. Light scattering and nickel(ii) spectrum thiolate → nickel(ii) charge-transfer peaks in the spectrum of the β-mercaptoethanol-urease complex. Biochim. Et Biophys. Acta (BBA)—Protein Struct. Mol. Enzym..

[22] Jørgensen C.K. (1955). Comparative crystal field studies of some ligands and the lowest singlet state of paramagnetic nickel(ii) complexes. Acta Chem. Scand..

[23] Christy A.A. (2014). The nature of silanol groups on the surfaces of silica, modified silica and some silica based materials. Adv. Mater. Res..

[24] Anderson J.H., Wickersheim K.A. (1964). Near infrared characterization of water and hydroxyl groups on silica surfaces. Surf. Sci..

[25] Klier K., Shen J.H., Zettlemoyer A.C. (1973). Water on silica and silicate surfaces. I. Partially hydrophobic silicas. J. Phys. Chem..

[26] Workman J., Weyer L. (2012). Practical Guide and Spectral Atlas for Interpretive Near-infrared Spectroscopy.

[27] Guidance for Industry: Q2b Validation of Analytical Procedures: Methodology. https://www.fda.gov/downloads/Drugs/GuidanceComplianceRegulatoryInformation/Guidances/UCM073384.pdf.

[28] Shabir G.A. (2004). A practical approach to validation of hplc methods under current good manufacturing practices. J. Valid. Technol..

[29] Kromidas S. (2011). Handbuch Validierung in der analytik.

[30] Geladi P., MacDougall D., Martens H. (1985). Linearization and scatter-correction for near-infrared reflectance spectra of meat. Appl. Spectrosc..

[31] Barnes R.J., Dhanoa M.S., Lister S.J. (1989). Standard normal variate transformation and de-trending of near-infrared diffuse reflectance spectra. Appl. Spectrosc..

[32] Savitzky A., Golay M.J.E. (1964). Smoothing and differentiation of data by simplified least squares procedures. Anal. Chem..

[33] Haaland D.M., Thomas E.V. (1988). Partial least-squares methods for spectral analyses. 1. Relation to other quantitative calibration methods and the extraction of qualitative information. Anal. Chem..

