# Supplementary material for: Direct Determination of Ni2+-Capacity of IMAC Materials Using Near-Infrared Spectroscopy
Source: Molecules. 2018 Nov 24;23(12):3072. doi: 10.3390/molecules23123072 (PMC6321272; doi:10.3390/molecules23123072)
Supplement: Supplementary file 1 [file molecules-23-03072-s001.zip › Supplementary Materials.pdf]

Supplementary Materials

Direct Determination of Ni^{2+}-capacity of IMAC materials using near-infrared spectroscopy

Christian G. Kirchler†1, Raphael Henn†1, Julia Modl[1], Felix Münzker[1], Tanja H. Baumgartner[1], Florian Meischl[1], Alexander Kehle[1], Günther K. Bonn[1,2] and Christian W. Huck[1,*]

[1] Institute of Analytical Chemistry and Radiochemistry, CCB-Center for Chemistry and Biomedicine, Innrain 80/82, 6020 Innsbruck, Austria; Analytische-Radiochemie@uibk.ac.at

[2] ADSI – Austrian Drug Screening Institute, Innrain 66a, 6020 Innsbruck, Austria; office@adsi.ac.at

† Authors contributed equally to this work.

* Correspondence: Christian.W.Huck@uibk.ac.at; Tel.: +43 512 507 57304

*address correspondence to:
Univ.-Prof.Mag.Dr. Christian Huck
Head of Spectroscopy Unit
Institute of Analytical Chemistry and Radiochemistry
CCB-Center for Chemistry and Biomedicine
Innrain 80/82
6020 Innsbruck
Austria
Mail: Christian.W.Huck@uibk.ac.at
Phone: +43 512 507 57304

Table of contents:

Table S1: List of synthesized IMAC materials. 3
Table S2: Calibration data for method validation of UV absorption spectroscopy day 1. 4
Table S3: Calibration data for method validation of UV absorption spectroscopy day 2. 5
Table S4: Calibration data for method validation of UV absorption spectroscopy day 3. 6
Table S5: Calibration data for method validation of UV absorption spectroscopy day 4. 7
Table S6: Calibration data for method validation of UV absorption spectroscopy day 5. 8
Table S7: Measured sample concentrations of the method validation of UV absorption spectroscopy day 1-2. 9
Table S8: Measured sample concentrations of the method validation of UV absorption spectroscopy day 3-4. 10
Table S9: Measured sample concentrations of the method validation of UV absorption spectroscopy day 5. 11
Table S10: Calibration data for method validation of AAS day 1. 12
Table S11: Calibration data for method validation of AAS day 2. 13
Table S12: Calibration data for method validation of AAS day 3. 14
Table S13: Calibration data for method validation of AAS day 4. 15
Table S14: Calibration data for method validation of AAS day 5. 16

Table S15: Measured sample concentrations of the method validation of AAS day 1-2.......... 17
Table S16: Measured sample concentrations of the method validation of AAS day 3-4.......... 18
Table S17: Measured sample concentrations of the method validation of AAS day 5. 19
Table S18: Calibration data for method validation of the Ni^{2+}-capacity determination by UV absorption spectroscopy day 1. 20
Table S19: Calibration data for method validation of the Ni^{2+}-capacity determination by UV absorption spectroscopy day 2. 21
Table S20: Calibration data for method validation of the Ni^{2+}-capacity determination by UV absorption spectroscopy day 3. 22
Table S21: Measured Ni^{2+}-capacity of the method validation by UV absorption spectroscopy day 1. 23
Table S22: Measured Ni^{2+}-capacity of the method validation by UV absorption spectroscopy day 2. 24
Table S23: Measured Ni^{2+}-capacity of the method validation by UV absorption spectroscopy day 3. 25

Table S1: List of synthesized IMAC materials.

IMAC-material No.	Porosity in Å	Particle size in	Ni^{2+}-capacity in (µmol/g)
**1**	35	40-63	357
**2**	35	40-63	408
**3**	35	40-63	405
**4**	60	20-45	338
**5**	60	20-45	411
**6**	60	20-45	377
**7**	60	20-45	354
**8**	60	30-200	436
**9**	60	35-70	400
**10**	60	40-63	379
**11**	60	40-63	409
**12**	60	40-63	369
**13**	60	40-63	359
**14**	60	63-200	363
**15**	60	63-200	263
**16**	60	63-200	317
**17**	60	63-200	339
**18**	60	200-500	444
**19**	100	63-200	342
**20**	100	63-200	280
**21**	100	63-200	274
**22**	100	63-200	276
**23**	150	35-70	377
**24**	150	35-70	317
**25**	150	35-70	246
**26**	150	35-70	247
**27**	150	70-200	352
**28**	250	40-63	311
**29**	250	40-63	214
**30**	250	40-63	352
**31**	250	40-63	277
**32**	1000	35-70	156

Table S2: Calibration data for method validation of UV absorption spectroscopy day 1.

**Calibration No.**	**1**	**2**	**3**	**4**
**Concentration in mol/L**	Absorbance	Absorbance	Absorbance	Absorbance
**0.005**	0.099	0.100	0.098	0.099
**0.005**	0.090	0.101	0.099	0.100
**0.005**	0.100	0.101	0.099	0.100
**0.013**	0.210	0.203	0.238	0.201
**0.013**	0.210	0.203	0.232	0.201
**0.013**	0.210	0.204	0.232	0.201
**0.021**	0.330	0.310	0.307	0.307
**0.021**	0.330	0.310	0.307	0.308
**0.021**	0.331	0.311	0.307	0.308
**0.029**	0.449	0.429	0.428	0.428
**0.029**	0.449	0.429	0.420	0.429
**0.029**	0.450	0.430	0.420	0.429
**0.037**	0.563	0.581	0.555	0.575
**0.037**	0.563	0.581	0.556	0.575
**0.037**	0.564	0.581	0.556	0.575
**0.045**	0.676	0.675	0.671	0.681
**0.045**	0.676	0.675	0.671	0.681
**0.045**	0.677	0.676	0.671	0.682
**Regression parameters for y=a+b*x**				
**a**	0.0234	0.0150	0.0295	0.0116
**b**	14.568	14.733	14.080	14.826
**R^2**	0.9998	0.9961	0.9949	0.9966
**LOD**	0.00028	0.0012	0.0014	0.0011
**LOQ**	0.0010	0.0042	0.0047	0.0039

Table S3: Calibration data for method validation of UV absorption spectroscopy day 2.

**Calibration No.**	**1**	**2**	**3**	**4**
**Concentration in mol/L**	Absorbance	Absorbance	Absorbance	Absorbance
**0.005**	0,103	0,102	0,101	0,102
**0.005**	0,103	0,103	0,101	0,102
**0.005**	0,103	0,104	0,106	0,102
**0.013**	0,212	0,210	0,206	0,207
**0.013**	0,212	0,211	0,207	0,207
**0.013**	0,212	0,212	0,207	0,212
**0.021**	0,325	0,324	0,315	0,325
**0.021**	0,325	0,324	0,315	0,325
**0.021**	0,325	0,353	0,319	0,326
**0.029**	0,443	0,449	0,438	0,440
**0.029**	0,443	0,449	0,439	0,441
**0.029**	0,444	0,450	0,440	0,442
**0.037**	0,570	0,581	0,571	0,571
**0.037**	0,571	0,581	0,572	0,571
**0.037**	0,571	0,582	0,572	0,572
**0.045**	0,683	0,685	0,699	0,683
**0.045**	0,684	0,685	0,700	0,684
**0.045**	0,688	0,686	0,703	0,684
**Regression parameters for y=a+b*x**				
**a**	0,0234	0,0244	0,0138	0,0215
**b**	14,658	14,780	15,027	14,686
**R^2**	0,9994	0,9985	0,9976	0,9993
**LOD**	0,00047	0,00075	0,00094	0,00052
**LOQ**	0,0017	0,0026	0,0033	0,0018

Table S4: Calibration data for method validation of UV absorption spectroscopy day 3.

**Calibration No.**	**1**	**2**	**3**	**4**
**Concentration in mol/L**	Absorbance	Absorbance	Absorbance	Absorbance
**0.005**	0,103	0,106	0,101	0,104
**0.005**	0,103	0,106	0,101	0,104
**0.005**	0,104	0,107	0,102	0,104
**0.013**	0,236	0,224	0,211	0,207
**0.013**	0,240	0,224	0,211	0,207
**0.013**	0,240	0,221	0,212	0,208
**0.021**	0,340	0,322	0,317	0,311
**0.021**	0,340	0,322	0,318	0,311
**0.021**	0,345	0,321	0,318	0,312
**0.029**	0,443	0,444	0,435	0,436
**0.029**	0,443	0,444	0,435	0,436
**0.029**	0,444	0,439	0,436	0,437
**0.037**	0,537	0,566	0,564	0,566
**0.037**	0,537	0,566	0,565	0,567
**0.037**	0,538	0,567	0,565	0,571
**0.045**	0,685	0,682	0,684	0,683
**0.045**	0,686	0,683	0,685	0,683
**0.045**	0,687	0,687	0,686	0,684
**Regression parameters for y=a+b*x**				
**a**	0,0425	0,0300	0,0202	0,0187
**b**	13,968	14,425	14,629	14,656
**R^2**	0,9952	0,9989	0,9988	0,9979
**LOD**	0,0013	0,00063	0,00067	0,00089
**LOQ**	0,0046	0,0022	0,0024	0,0031

Table S5: Calibration data for method validation of UV absorption spectroscopy day 4.

**Calibration No.**	**1**	**2**	**3**	**4**
**Concentration in mol/L**	Absorbance	Absorbance	Absorbance	Absorbance
**0.005**	0,103	0,103	0,105	0,098
**0.005**	0,103	0,104	0,106	0,099
**0.005**	0,104	0,105	0,106	0,099
**0.013**	0,207	0,209	0,202	0,210
**0.013**	0,208	0,209	0,202	0,211
**0.013**	0,209	0,214	0,203	0,211
**0.021**	0,324	0,317	0,316	0,310
**0.021**	0,325	0,318	0,316	0,311
**0.021**	0,325	0,318	0,320	0,311
**0.029**	0,442	0,445	0,435	0,430
**0.029**	0,443	0,445	0,436	0,430
**0.029**	0,443	0,446	0,436	0,435
**0.037**	0,568	0,567	0,566	0,560
**0.037**	0,569	0,567	0,566	0,561
**0.037**	0,571	0,568	0,567	0,562
**0.045**	0,682	0,683	0,680	0,676
**0.045**	0,683	0,683	0,680	0,676
**0.045**	0,684	0,684	0,681	0,677
**Regression parameters for y=a+b*x**				
**a**	0,0224	0,0225	0,0200	0,0190
**b**	14,644	14,623	14,585	14,501
**R^2**	0,9993	0,9990	0,9983	0,9985
**LOD**	0,00050	0,00062	0,00080	0,00074
**LOQ**	0,0018	0,0022	0,0028	0,0026

Table S6: Calibration data for method validation of UV absorption spectroscopy day 5.

**Calibration No.**	**1**	**2**	**3**	**4**
**Concentration in mol/L**	Absorbance	Absorbance	Absorbance	Absorbance
**0.005**	0,113	0,114	0,111	0,111
**0.005**	0,113	0,115	0,111	0,111
**0.005**	0,114	0,116	0,116	0,112
**0.013**	0,211	0,207	0,203	0,201
**0.013**	0,211	0,208	0,204	0,202
**0.013**	0,212	0,209	0,205	0,207
**0.021**	0,337	0,316	0,311	0,309
**0.021**	0,337	0,317	0,311	0,309
**0.021**	0,338	0,318	0,312	0,310
**0.029**	0,436	0,435	0,429	0,429
**0.029**	0,436	0,435	0,429	0,429
**0.029**	0,437	0,436	0,430	0,430
**0.037**	0,567	0,574	0,563	0,564
**0.037**	0,568	0,574	0,564	0,565
**0.037**	0,568	0,575	0,565	0,565
**0.045**	0,681	0,682	0,678	0,677
**0.045**	0,682	0,682	0,679	0,678
**0.045**	0,683	0,683	0,680	0,679
**Regression parameters for y=a+b*x**				
**a**	0,0332	0,0267	0,0236	0,0222
**b**	14,326	14,479	14,392	14,419
**R^2**	0,9985	0,9967	0,9965	0,9964
**LOD**	0,00074	0,0011	0,0011	0,0011
**LOQ**	0,0026	0,0038	0,0039	0,0040

Table S7: Measured sample concentrations of the method validation of UV absorption spectroscopy day 1-2.

**Measurement**	**1**		**2**		**3**		**4**	
**Day**	Absorbance	Concentration in mmol/L	Absorbance	Concentration in mmol/L	Absorbance	Concentration in mmol/L	Absorbance	Concentration in mmol/L
**1**	0,178	10,6	0,180	11,2	0,190	11,4	0,182	11,5
	0,178	10,6	0,180	11,2	0,191	11,5	0,182	11,5
	0,179	10,7	0,181	11,3	0,191	11,5	0,181	11,4
	0,355	22,8	0,355	23,1	0,340	22,1	0,341	22,2
	0,355	22,8	0,355	23,1	0,341	22,1	0,341	22,2
	0,355	22,8	0,355	23,1	0,341	22,1	0,342	22,3
	0,522	34,2	0,545	36,0	0,524	35,1	0,533	35,2
	0,522	34,2	0,545	36,0	0,525	35,2	0,533	35,2
	0,522	34,2	0,545	36,0	0,525	35,2	0,533	35,2
**2**	0,181	10,8	0,180	10,5	0,180	11,1	0,186	11,2
	0,181	10,8	0,181	10,6	0,181	11,1	0,186	11,2
	0,182	10,8	0,182	10,7	0,181	11,1	0,187	11,3
	0,344	21,9	0,366	23,1	0,369	23,6	0,346	22,1
	0,345	21,9	0,367	23,2	0,369	23,6	0,347	22,2
	0,346	22,0	0,367	23,2	0,370	23,7	0,347	22,2
	0,525	34,2	0,539	34,8	0,524	34,0	0,511	33,3
	0,525	34,2	0,539	34,8	0,524	34,0	0,511	33,3
	0,526	34,3	0,540	34,9	0,525	34,0	0,512	33,4

Table S8: Measured sample concentrations of the method validation of UV absorption spectroscopy day 3-4.

**Measurement**	**1**		**2**		**3**		**4**	
**Day**	Absorbance	Concentration in mmol/L	Absorbance	Concentration in mmol/L	Absorbance	Concentration in mmol/L	Absorbance	Concentration in mmol/L
**3**	0,180	9,84	0,180	10,4	0,177	10,7	0,178	10,9
	0,181	9,91	0,181	10,5	0,177	10,7	0,179	10,9
	0,182	9,99	0,181	10,5	0,178	10,8	0,180	11,0
	0,343	21,5	0,342	21,6	0,340	21,9	0,340	21,9
	0,343	21,5	0,343	21,7	0,341	21,9	0,345	22,3
	0,344	21,6	0,347	22,0	0,341	21,9	0,345	22,3
	0,525	34,5	0,546	35,8	0,523	34,4	0,522	34,3
	0,525	34,5	0,546	35,8	0,524	34,4	0,523	34,4
	0,526	34,6	0,549	36,0	0,528	34,7	0,523	34,4
**4**	0,180	10,8	0,180	10,8	0,178	10,8	0,174	10,7
	0,180	10,8	0,181	10,8	0,179	10,9	0,175	10,8
	0,185	11,1	0,182	10,9	0,180	11,0	0,179	11,0
	0,343	21,9	0,346	22,1	0,341	22,0	0,337	21,9
	0,343	21,9	0,346	22,1	0,341	22,0	0,338	22,0
	0,344	22,0	0,347	22,2	0,346	22,4	0,338	22,0
	0,526	34,4	0,525	34,4	0,523	34,5	0,518	34,4
	0,526	34,4	0,525	34,4	0,524	34,6	0,519	34,5
	0,527	34,5	0,526	34,4	0,524	34,6	0,520	34,6

Table S9: Measured sample concentrations of the method validation of UV absorption spectroscopy day 5.

**Measurement**	**1**		**2**		**3**		**4**	
**Day**	Absorbance	Concentration in mmol/L	Absorbance	Concentration in mmol/L	Absorbance	Concentration in mmol/L	Absorbance	Concentration in mmol/L
**5**	0,178	10,1	0,176	10,3	0,181	10,9	0,176	10,7
	0,179	10,2	0,176	10,3	0,182	11,0	0,176	10,7
	0,180	10,2	0,177	10,4	0,182	11,0	0,177	10,7
	0,341	21,5	0,344	21,9	0,341	22,1	0,339	22,0
	0,341	21,5	0,344	21,9	0,342	22,1	0,339	22,0
	0,342	21,6	0,345	22,0	0,342	22,1	0,340	22,0
	0,526	34,4	0,519	34,0	0,521	34,6	0,520	34,5
	0,527	34,5	0,520	34,1	0,522	34,6	0,520	34,5
	0,532	34,8	0,521	34,1	0,523	34,7	0,521	34,6

Table S10: Calibration data for method validation of AAS day 1.

**Calibration No.**	**1**	**2**	**3**	**4**
**Concentration in mol/L**	Absorbance	Absorbance	Absorbance	Absorbance
**$3{,}387*10^{-5}$**	0,051	0,061	0,045	0,059
**$3{,}387*10^{-5}$**	0,042	0,055	0,049	0,042
**$3{,}387*10^{-5}$**	0,061	0,052	0,055	0,049
**$8{,}520*10^{-5}$**	0,113	0,115	0,111	0,111
**$8{,}520*10^{-5}$**	0,114	0,120	0,119	0,122
**$8{,}520*10^{-5}$**	0,121	0,119	0,121	0,119
**$1{,}191*10^{-4}$**	0,163	0,150	0,161	0,160
**$1{,}191*10^{-4}$**	0,166	0,169	0,155	0,166
**$1{,}191*10^{-4}$**	0,148	0,152	0,162	0,170
**$1{,}535*10^{-4}$**	0,202	0,204	0,203	0,199
**$1{,}535*10^{-4}$**	0,204	0,203	0,217	0,198
**$1{,}535*10^{-4}$**	0,194	0,202	0,205	0,203
**$2{,}043*10^{-4}$**	0,251	0,258	0,251	0,254
**$2{,}043*10^{-4}$**	0,253	0,268	0,262	0,266
**$2{,}043*10^{-4}$**	0,246	0,269	0,250	0,271
**$2{,}560*10^{-4}$**	0,303	0,312	0,304	0,315
**$2{,}560*10^{-4}$**	0,299	0,309	0,316	0,309
**$2{,}560*10^{-4}$**	0,300	0,308	0,313	0,315
**Regression parameters for y=a+b*x**				
**a**	0,0203	0,0194	0,0169	0,0162
**b**	1120,9	1164,8	1172,1	1188,1
**R^2**	0,9916	0,9938	0,9918	0,9929
**LOD**	$9{,}6*10^{-6}$	$8{,}3*10^{-6}$	$9{,}6*10^{-6}$	$8{,}9*10^{-6}$
**LOQ**	$3{,}3*10^{-5}$	$2{,}8*10^{-5}$	$3{,}2*10^{-5}$	$3{,}0*10^{-5}$

Table S11: Calibration data for method validation of AAS day 2.

**Calibration No.**	**1**	**2**	**3**	**4**
**Concentration in mol/L**	Absorbance	Absorbance	Absorbance	Absorbance
**$3,387*10^{-5}$**	0,055	0,044	0,056	0,049
**$3,387*10^{-5}$**	0,048	0,059	0,047	0,047
**$3,387*10^{-5}$**	0,050	0,062	0,053	0,040
**$8,520*10^{-5}$**	0,135	0,140	0,132	0,106
**$8,520*10^{-5}$**	0,120	0,135	0,126	0,121
**$8,520*10^{-5}$**	0,122	0,126	0,125	0,111
**$1,191*10^{-4}$**	0,164	0,180	0,165	0,149
**$1,191*10^{-4}$**	0,158	0,187	0,165	0,153
**$1,191*10^{-4}$**	0,161	0,179	0,168	0,160
**$1,535*10^{-4}$**	0,221	0,232	0,201	0,193
**$1,535*10^{-4}$**	0,199	0,230	0,205	0,194
**$1,535*10^{-4}$**	0,213	0,241	0,208	0,191
**$2,043*10^{-4}$**	0,250	0,294	0,266	0,237
**$2,043*10^{-4}$**	0,254	0,306	0,279	0,232
**$2,043*10^{-4}$**	0,251	0,289	0,271	0,227
**$2,560*10^{-4}$**	0,309	0,347	0,325	0,287
**$2,560*10^{-4}$**	0,302	0,352	0,313	0,287
**$2,560*10^{-4}$**	0,305	0,353	0,316	0,289
**Regression parameters for y=a+b*x**				
**a**	0,0242	0,0186	0,0199	0,0192
**b**	1127,2	1338,4	1198,1	1067,5
**R^2**	0,9863	0,9911	0,9929	0,9892
**LOD**	$1,2*10^{-5}$	$1,0*10^{-5}$	$8,9*10^{-6}$	$1,1*10^{-5}$
**LOQ**	$4,1*10^{-5}$	$3,4*10^{-5}$	$3,0*10^{-5}$	$3,7*10^{-5}$

Table S12: Calibration data for method validation of AAS day 3.

**Calibration No.**	**1**	**2**	**3**	**4**
**Concentration in mol/L**	Absorbance	Absorbance	Absorbance	Absorbance
**$3,387*10^{-5}$**	0,054	0,052	0,041	0,064
**$3,387*10^{-5}$**	0,048	0,061	0,059	0,045
**$3,387*10^{-5}$**	0,052	0,046	0,043	0,060
**$8,520*10^{-5}$**	0,130	0,122	0,127	0,132
**$8,520*10^{-5}$**	0,138	0,120	0,127	0,143
**$8,520*10^{-5}$**	0,125	0,122	0,141	0,131
**$1,191*10^{-4}$**	0,180	0,172	0,203	0,195
**$1,191*10^{-4}$**	0,182	0,162	0,185	0,188
**$1,191*10^{-4}$**	0,178	0,176	0,171	0,202
**$1,535*10^{-4}$**	0,202	0,201	0,237	0,236
**$1,535*10^{-4}$**	0,209	0,193	0,231	0,229
**$1,535*10^{-4}$**	0,199	0,205	0,223	0,229
**$2,043*10^{-4}$**	0,269	0,265	0,281	0,294
**$2,043*10^{-4}$**	0,260	0,261	0,284	0,298
**$2,043*10^{-4}$**	0,254	0,263	0,285	0,290
**$2,560*10^{-4}$**	0,306	0,301	0,341	0,354
**$2,560*10^{-4}$**	0,311	0,313	0,338	0,352
**$2,560*10^{-4}$**	0,308	0,302	0,344	0,353
**Regression parameters for y=a+b*x**				
**a**	0,0289	0,0236	0,0184	0,0228
**b**	1129,1	1139,5	1302,9	1324,5
**R^2**	0,9821	0,9888	0,9826	0,9896
**LOD**	$1,4*10^{-5}$	$1,1*10^{-5}$	$1,4*10^{-5}$	$1,1*10^{-5}$
**LOQ**	$4,7*10^{-5}$	$3,7*10^{-5}$	$4,6*10^{-5}$	$3,6*10^{-5}$

Table S13: Calibration data for method validation of AAS day 4.

**Calibration No.**	**1**	**2**	**3**	**4**
**Concentration in mol/L**	Absorbance	Absorbance	Absorbance	Absorbance
**$3,387*10^{-5}$**	0,051	0,051	0,051	0,051
**$3,387*10^{-5}$**	0,059	0,056	0,057	0,051
**$3,387*10^{-5}$**	0,053	0,060	0,064	0,044
**$8,520*10^{-5}$**	0,135	0,136	0,138	0,113
**$8,520*10^{-5}$**	0,146	0,142	0,132	0,110
**$8,520*10^{-5}$**	0,137	0,141	0,128	0,116
**$1,191*10^{-4}$**	0,175	0,190	0,181	0,149
**$1,191*10^{-4}$**	0,184	0,181	0,172	0,146
**$1,191*10^{-4}$**	0,175	0,194	0,178	0,154
**$1,535*10^{-4}$**	0,214	0,240	0,218	0,184
**$1,535*10^{-4}$**	0,221	0,236	0,216	0,187
**$1,535*10^{-4}$**	0,219	0,241	0,220	0,192
**$2,043*10^{-4}$**	0,289	0,296	0,280	0,251
**$2,043*10^{-4}$**	0,283	0,303	0,293	0,246
**$2,043*10^{-4}$**	0,294	0,302	0,285	0,243
**$2,560*10^{-4}$**	0,338	0,360	0,340	0,298
**$2,560*10^{-4}$**	0,328	0,359	0,344	0,307
**$2,560*10^{-4}$**	0,332	0,362	0,344	0,294
**Regression parameters for y=a+b*x**				
**a**	0,0244	0,0202	0,0202	0,0142
**b**	1249,7	1364,5	1282,1	1127,4
**R^2**	0,9890	0,9935	0,9961	0,9972
**LOD**	$1,1*10^{-5}$	$8,5*10^{-6}$	$6,6*10^{-6}$	$5,6*10^{-6}$
**LOQ**	$3,7*10^{-5}$	$2,9*10^{-5}$	$2,3*10^{-5}$	$1,9*10^{-5}$

Table S14: Calibration data for method validation of AAS day 5.

**Calibration No.**	**1**	**2**	**3**	**4**
**Concentration in mol/L**	Absorbance	Absorbance	Absorbance	Absorbance
**3,387*10^{-5}**	0,028	0,065	0,048	0,035
**3,387*10^{-5}**	0,034	0,050	0,056	0,048
**3,387*10^{-5}**	0,048	0,059	0,049	0,055
**8,520*10^{-5}**	0,090	0,130	0,121	0,101
**8,520*10^{-5}**	0,090	0,135	0,125	0,105
**8,520*10^{-5}**	0,092	0,134	0,123	0,110
**1,191*10^{-4}**	0,121	0,174	0,172	0,160
**1,191*10^{-4}**	0,128	0,179	0,173	0,155
**1,191*10^{-4}**	0,113	0,183	0,167	0,157
**1,535*10^{-4}**	0,158	0,220	0,234	0,205
**1,535*10^{-4}**	0,145	0,230	0,216	0,203
**1,535*10^{-4}**	0,156	0,223	0,226	0,206
**2,043*10^{-4}**	0,197	0,273	0,283	0,251
**2,043*10^{-4}**	0,205	0,272	0,288	0,258
**2,043*10^{-4}**	0,205	0,289	0,290	0,258
**2,560*10^{-4}**	0,248	0,332	0,329	0,306
**2,560*10^{-4}**	0,246	0,339	0,346	0,291
**2,560*10^{-4}**	0,242	0,335	0,332	0,306
**Regression parameters for y=a+b*x**				
**a**	0,0082	0,0253	0,0136	0,0119
**b**	938,6	1239,3	1304,5	1172,2
**R^2**	0,9932	0,9921	0,9908	0,9876
**LOD**	8,7*10^{-6}	9,4*10^{-6}	1,0*10^{-5}	1,2*10^{-5}
**LOQ**	3,0*10^{-5}	3,2*10^{-5}	3,4*10^{-5}	3,9*10^{-5}

Table S15: Measured sample concentrations of the method validation of AAS day 1-2.

**Measurement**	**1**		**2**		**3**		**4**	
**Day**	Absorbance	Concentration in mmol/L	Absorbance	Concentration in mmol/L	Absorbance	Concentration in mmol/L	Absorbance	Concentration in mmol/L
**1**	0,090	0,0621	0,105	0,0735	0,099	0,0701	0,100	0,0705
	0,084	0,0568	0,091	0,0615	0,101	0,0718	0,104	0,0739
	0,088	0,0604	0,113	0,0804	0,092	0,0641	0,105	0,0747
	0,168	0,1317	0,186	0,1430	0,174	0,1341	0,191	0,1471
	0,164	0,1282	0,175	0,1336	0,172	0,1324	0,191	0,1471
	0,164	0,1282	0,181	0,1387	0,164	0,1255	0,201	0,1555
	0,230	0,1870	0,231	0,1817	0,225	0,1776	0,242	0,1900
	0,235	0,1915	0,228	0,1791	0,236	0,1870	0,241	0,1892
	0,234	0,1906	0,236	0,1860	0,232	0,1835	0,236	0,1850
**2**	0,108	0,0743	0,103	0,0630	0,099	0,0660	0,080	0,0570
	0,083	0,0521	0,111	0,0690	0,095	0,0626	0,092	0,0682
	0,097	0,0646	0,108	0,0668	0,098	0,0651	0,079	0,0561
	0,180	0,1382	0,205	0,1392	0,176	0,1302	0,176	0,1469
	0,184	0,1417	0,206	0,1400	0,183	0,1361	0,162	0,1338
	0,177	0,1355	0,189	0,1273	0,181	0,1344	0,178	0,1488
	0,206	0,1613	0,252	0,1744	0,239	0,1828	0,215	0,1835
	0,214	0,1683	0,260	0,1803	0,246	0,1887	0,208	0,1769
	0,203	0,1586	0,254	0,1759	0,239	0,1828	0,212	0,1806

Table S16: Measured sample concentrations of the method validation of AAS day 3-4.

**Measurement**	**1**		**2**		**3**		**4**	
**Day**	Absorbance	Concentration in mmol/L	Absorbance	Concentration in mmol/L	Absorbance	Concentration in mmol/L	Absorbance	Concentration in mmol/L
**3**	0,110	0,0719	0,103	0,0697	0,115	0,0741	0,108	0,0643
	0,104	0,0666	0,098	0,0653	0,128	0,0841	0,116	0,0704
	0,108	0,0701	0,101	0,0679	0,129	0,0849	0,115	0,0696
	0,191	0,1436	0,189	0,1451	0,221	0,1555	0,198	0,1323
	0,189	0,1418	0,179	0,1364	0,202	0,1409	0,192	0,1278
	0,185	0,1383	0,174	0,1320	0,197	0,1371	0,195	0,1300
	0,246	0,1923	0,237	0,1873	0,259	0,1847	0,268	0,1851
	0,241	0,1879	0,242	0,1917	0,261	0,1862	0,248	0,1700
	0,235	0,1826	0,231	0,1820	0,263	0,1877	0,264	0,1821
**4**	0,098	0,0589	0,115	0,0695	0,091	0,0552	0,102	0,0779
	0,107	0,0661	0,115	0,0695	0,100	0,0622	0,096	0,0726
	0,106	0,0653	0,120	0,0732	0,106	0,0669	0,093	0,0699
	0,193	0,1349	0,213	0,1413	0,181	0,1254	0,184	0,1507
	0,188	0,1309	0,215	0,1428	0,189	0,1316	0,178	0,1453
	0,185	0,1285	0,209	0,1384	0,190	0,1324	0,184	0,1507
	0,242	0,1741	0,276	0,1875	0,244	0,1745	0,224	0,1861
	0,250	0,1805	0,273	0,1853	0,250	0,1792	0,220	0,1826
	0,246	0,1773	0,272	0,1846	0,240	0,1714	0,222	0,1844

Table S17: Measured sample concentrations of the method validation of AAS day 5.

**Measurement**	**1**		**2**		**3**		**4**	
**Day**	Absorbance	Concentration in mmol/L	Absorbance	Concentration in mmol/L	Absorbance	Concentration in mmol/L	Absorbance	Concentration in mmol/L
**5**	0,062	0,0573	0,109	0,0676	0,092	0,0601	0,092	0,0683
	0,070	0,0659	0,115	0,0724	0,097	0,0640	0,093	0,0692
	0,078	0,0744	0,115	0,0724	0,104	0,0693	0,099	0,0743
	0,131	0,1309	0,201	0,1418	0,213	0,1529	0,184	0,1468
	0,136	0,1362	0,207	0,1466	0,203	0,1452	0,176	0,1400
	0,132	0,1319	0,208	0,1474	0,200	0,1429	0,176	0,1400
	0,172	0,1745	0,274	0,2007	0,252	0,1828	0,239	0,1937
	0,175	0,1777	0,263	0,1918	0,256	0,1858	0,234	0,1895
	0,168	0,1703	0,268	0,1959	0,253	0,1835	0,233	0,1886

Table S18: Calibration data for method validation of the Ni^{2+}-capacity determination by UV absorption spectroscopy day 1.

**Calibration No.**	**1**	**2**	**3**
**Concentration in mol/L**	Absorbance	Absorbance	Absorbance
**0.005**	0,101	0,099	0,101
**0.005**	0,101	0,099	0,101
**0.005**	0,101	0,100	0,102
**0.013**	0,216	0,206	0,209
**0.013**	0,217	0,206	0,209
**0.013**	0,218	0,207	0,208
**0.021**	0,320	0,328	0,312
**0.021**	0,321	0,328	0,310
**0.021**	0,326	0,329	0,314
**0.029**	0,442	0,445	0,434
**0.029**	0,443	0,446	0,435
**0.029**	0,443	0,447	0,435
**0.037**	0,565	0,561	0,560
**0.037**	0,565	0,561	0,561
**0.037**	0,566	0,562	0,562
**0.045**	0,677	0,675	0,675
**0.045**	0,678	0,675	0,675
**0.045**	0,678	0,676	0,676
**Regression parameters for y=a+b*x**			
**a**	0,0262	0,0234	0,0206
**b**	14,460	14,510	14,463
**R^2**	0,9996	0,9998	0,9987
**LOD**	0,00041	0,00028	0,00068
**LOQ**	0,0015	0,0010	0,0024

Table S19: Calibration data for method validation of the Ni^{2+}-capacity determination by UV absorption spectroscopy day 2.

**Calibration No.**	**1**	**2**	**3**
**Concentration in mol/L**	Absorbance	Absorbance	Absorbance
**0.005**	0,099	0,101	0,101
**0.005**	0,099	0,101	0,101
**0.005**	0,100	0,106	0,106
**0.013**	0,218	0,209	0,207
**0.013**	0,219	0,210	0,208
**0.013**	0,220	0,210	0,212
**0.021**	0,346	0,349	0,318
**0.021**	0,347	0,349	0,319
**0.021**	0,353	0,354	0,319
**0.029**	0,443	0,430	0,446
**0.029**	0,444	0,444	0,446
**0.029**	0,448	0,444	0,447
**0.037**	0,577	0,571	0,568
**0.037**	0,577	0,572	0,569
**0.037**	0,582	0,573	0,573
**0.045**	0,677	0,683	0,685
**0.045**	0,678	0,684	0,686
**0.045**	0,678	0,685	0,690
**Regression parameters for y=a+b*x**			
**a**	0,0316	0,0286	0,0200
**b**	14,525	14,580	14,758
**R^2**	0,9983	0,9977	0,9989
**LOD**	0,00079	0,00092	0,00063
**LOQ**	0,0028	0,0032	0,0022

Table S20: Calibration data for method validation of the Ni^{2+}-capacity determination by UV absorption spectroscopy day 3.

**Calibration No.**	**1**	**2**	**3**
**Concentration in mol/L**	Absorbance	Absorbance	Absorbance
**0.005**	0,104	0,102	0,105
**0.005**	0,104	0,102	0,105
**0.005**	0,109	0,108	0,106
**0.013**	0,210	0,213	0,217
**0.013**	0,211	0,213	0,218
**0.013**	0,216	0,214	0,218
**0.021**	0,340	0,327	0,325
**0.021**	0,341	0,327	0,325
**0.021**	0,341	0,328	0,326
**0.029**	0,448	0,439	0,441
**0.029**	0,449	0,440	0,441
**0.029**	0,449	0,440	0,445
**0.037**	0,572	0,569	0,576
**0.037**	0,573	0,569	0,576
**0.037**	0,574	0,570	0,577
**0.045**	0,685	0,689	0,686
**0.045**	0,686	0,689	0,686
**0.045**	0,687	0,690	0,687
**Regression parameters for y=a+b*x**			
**a**	0,0291	0,0238	0,0263
**b**	14,613	14,668	14,636
**R^2**	0,9995	0,9991	0,9989
**LOD**	0,00043	0,00056	0,00062
**LOQ**	0,0016	0,0020	0,0022

Table S21: Measured Ni^{2+}-capacity of the method validation by UV absorption spectroscopy day 1.

**Measurement No.**		1			2				3			
**IMAC**	m in mg	Absorbance	Concentration in mmol/L	Ni^{2+}-capacity in (µmol/g)	m / mg	Absorbance	Concentration in mmol/L	Ni^{2+}-capacity in (µmol/g)	m / mg	Absorbance	Concentration in mmol/L	Ni^{2+}-capacity in (µmol/g)
**1**	99,90	0,130	7,18	359	100,32	0,123	6,87	342	99,87	0,129	7,50	375
		0,131	7,25	363		0,123	6,87	342		0,129	7,50	375
		0,136	7,60	380		0,124	6,94	346		0,130	7,56	379
**2**	100,45	0,136	7,60	378	100,26	0,118	6,52	325	100,36	0,117	6,67	332
		0,136	7,60	378		0,118	6,52	325		0,118	6,74	336
		0,141	7,94	395		0,119	6,59	329		0,119	6,80	339
**3**	99,62	0,106	5,52	277	100,35	0,107	5,76	287	99,85	0,111	6,25	313
		0,107	5,59	281		0,107	5,76	287		0,112	6,32	316
		0,108	5,66	284		0,108	5,83	291		0,116	6,60	330
**4**	99,84	0,129	7,11	356	100,08	0,123	6,87	343	100,05	0,123	7,08	354
		0,130	7,18	360		0,124	6,94	346		0,124	7,15	357
		0,130	7,18	360		0,124	6,94	346		0,124	7,15	357
**5**	100,07	0,140	7,87	393	99,90	0,140	8,04	402	100,18	0,137	8,05	402
		0,140	7,87	393		0,141	8,11	406		0,138	8,12	405
		0,145	8,22	411		0,142	8,18	409		0,143	8,46	422
**6**	100,03	0,139	7,80	390	99,75	0,149	8,66	434	100,17	0,137	8,05	402
		0,139	7,80	390		0,149	8,66	434		0,137	8,05	402
		0,140	7,87	393		0,150	8,73	437		0,138	8,12	405

Table S22: Measured Ni^{2+}-capacity of the method validation by UV absorption spectroscopy day 2.

**Measurement No.**		1			2				3			
**IMAC**	m in mg	Absorbance	Concentration in mmol/L	Ni^{2+}-capacity in (µmol/g)	m / mg	Absorbance	Concentration in mmol/L	Ni^{2+}-capacity in (µmol/g)	m / mg	Absorbance	Concentration in mmol/L	Ni^{2+}-capacity in (µmol/g)
**1**	100,36	0,123	6,29	314	99,81	0,129	6,89	345	100,30	0,130	7,45	372
		0,124	6,36	317		0,129	6,89	345		0,131	7,52	375
		0,125	6,43	320		0,130	6,96	349		0,136	7,86	392
**2**	99,76	0,120	6,09	305	100,01	0,119	6,20	310	100,09	0,119	6,71	335
		0,120	6,09	305		0,120	6,27	314		0,120	6,78	339
		0,121	6,16	308		0,121	6,34	317		0,121	6,84	342
**3**	100,39	0,121	6,16	307	99,97	0,113	5,79	290	100,12	0,109	6,03	301
		0,121	6,16	307		0,114	5,86	293		0,110	6,10	305
		0,122	6,22	310		0,114	5,86	293		0,114	6,37	318
**4**	100,14	0,128	6,64	331	100,34	0,126	6,68	333	100,01	0,126	7,18	359
		0,129	6,71	335		0,126	6,68	333		0,126	7,18	359
		0,133	6,98	349		0,127	6,75	336		0,131	7,52	376
**5**	100,16	0,144	7,74	386	100,42	0,153	8,54	425	100,32	0,143	8,34	415
		0,145	7,81	390		0,153	8,54	425		0,144	8,40	419
		0,145	7,81	390		0,154	8,60	428		0,145	8,47	422
**6**	99,86	0,145	7,81	391	99,90	0,150	8,33	417	100,16	0,144	8,40	419
		0,145	7,81	391		0,150	8,33	417		0,144	8,40	419
		0,146	7,88	394		0,151	8,40	420		0,144	8,40	419

Table S23: Measured Ni^{2+}-capacity of the method validation by UV absorption spectroscopy day 3.

**Measurement No.**		**1**			**2**				**3**			
**IMAC**	m in mg	Absorbance	Concentration in mmol/L	Ni^{2+}-capacity in (µmol/g)	m / mg	Absorbance	Concentration in mmol/L	Ni^{2+}-capacity in (µmol/g)	m / mg	Absorbance	Concentration in mmol/L	Ni^{2+}-capacity in (µmol/g)
**1**	99,78	0,127	6,70	336	99,93	0,125	6,90	345	99,99	0,134	7,36	368
		0,127	6,70	336		0,126	6,97	349		0,135	7,43	371
		0,132	7,04	353		0,127	7,04	352		0,140	7,77	388
**2**	100,22	0,120	6,22	310	100,06	0,120	6,56	328	100,47	0,122	6,54	325
		0,121	6,29	314		0,121	6,63	331		0,122	6,54	325
		0,122	6,36	317		0,122	6,69	335		0,123	6,61	329
**3**	98,84	0,117	6,02	304	99,78	0,111	5,94	298	99,95	0,118	6,26	313
		0,118	6,09	308		0,111	5,94	298		0,118	6,26	313
		0,123	6,43	325		0,112	6,01	301		0,119	6,33	317
**4**	100,08	0,123	6,43	321	99,94	0,123	6,76	338	100,18	0,132	7,22	360
		0,124	6,50	325		0,123	6,76	338		0,133	7,29	364
		0,129	6,84	342		0,124	6,83	342		0,134	7,36	367
**5**	99,78	0,149	8,21	411	99,90	0,154	8,88	444	100,37	0,148	8,31	414
		0,149	8,21	411		0,153	8,81	441		0,149	8,38	418
		0,150	8,28	415		0,150	8,60	431		0,149	8,38	418
**6**	100,40	0,136	7,32	364	100,33	0,137	7,72	385	100,10	0,137	7,56	378
		0,136	7,32	364		0,137	7,72	385		0,137	7,56	378
		0,141	7,66	381		0,138	7,79	388		0,138	7,63	381
